# Rib construct for severe spinal deformity in young children: a 3-part investigation of biomechanical, animal, and clinical case data

**DOI:** 10.1016/j.xnsj.2025.100776

**Published:** 2025-07-30

**Authors:** Daniel J. Bonthius, Richard H. Gross, Mohammed A. Alshareef, Gregory J. Wright, Shuchun Sun, Yongren Wu, Hai Yao

**Affiliations:** a Department of Bioengineering, Clemson University, Clemson, SC, United States; b College of Medicine, Medical University of South Carolina, Charleston, SC, United States; c Department of Neurosurgery, Medical University of South Carolina, Charleston, SC, United States; d Department of Orthopedics, Medical University of South Carolina, Charleston, SC, United States

**Keywords:** Spinal deformity, Hyperkyphosis, Rib construct, Pedicle screw, Growth modulation, Retrospective clinical study, Mechanical testing, Animal model

## Abstract

**Background:**

Early-onset spinal deformity (EOSD), occurring before age 10, requires surgical techniques that accommodate spinal growth. Traditional intra-spinal methods like growing rods have high complication rates. The rib construct is an alternative technique that uses rib-based fixation for correcting EOSD. The objective of this study is to evaluate its performance.

**Methods:**

Biomechanical bending and torsional tests on 20 harvested pig spines compared the pull-out and twisting forces between the rib construct and pedicle screw. For the animal study, hyperkyphosis was induced in 6 immature pigs and subsequently corrected using the rib construct; radiographic and histological evaluations assessed the correction outcomes. Retrospective clinical data on 14 patients (8 male, 6 female) treated with the rib construct for severe nonidiopathic spinal deformity were studied including diagnosis, age at index surgery, length of follow-up, T-score bone density, complication rates, procedure time, operative blood loss, and radiographic outcomes.

**Results:**

Biomechanical testing studies demonstrated that the rib construct was less prone to proximal fixation failure and less stiff compared to pedicle screws. Animal model studies demonstrated improvement in spinal alignment in hyperkyphotic pigs instrumented with the rib construct. Finally, clinical study outcomes demonstrated excellent deformity correction with the rib construct and a reduction in serious complications compared to other techniques.

**Conclusions:**

The rib construct effectively corrects spinal deformity through growth modulation while supporting spinal growth and pulmonary development. It substantially reduces the incidence of severe complications commonly associated with EOSD treatments and is particularly beneficial in cases involving hyperkyphosis and/or osteoporosis.

## Introduction

**Fig. 1:**
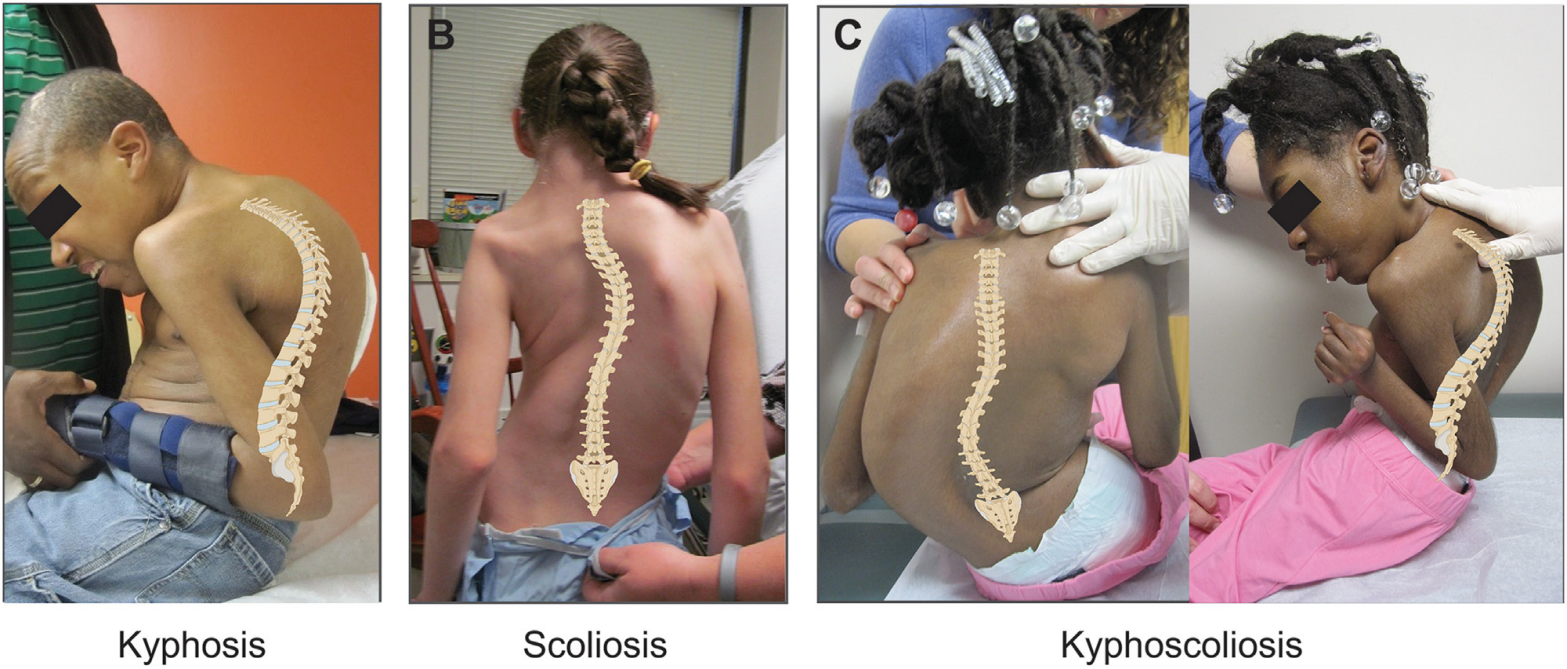
**Spinal deformity.** A) A 20-year-old male with severe hyperkyphosis. B) An 11-year-old girl with scoliosis and a substantial rib hump. C) A 12-year-old girl with kyphoscoliosis.

Pediatric spinal deformity is a common pathology of childhood in which the spine curves abnormally. This can include deformities in the sagittal plane (kyphosis), coronal plane (scoliosis), or both (kyphoscoliosis) (Fig. 1A-C), and affects 1-8 percent of all children worldwide [1]. Early-onset spinal deformity (EOSD) is a subset of pediatric spinal deformity that begins before the age of 10 years and affects 0.2-0.6 percent of children [2]. Left untreated, its consequences include debilitating deformity, neurological deficits, respiratory failure, cardiac disease, and early mortality [3]. Although less common than adolescent deformity, EOSD is 1 of the most difficult orthopedic conditions to treat because surgical intervention must accommodate growth of the developing spine, as premature spinal fusion techniques lead to stunted spinal growth and pulmonary insufficiency [4,5].

**Fig. 2:**
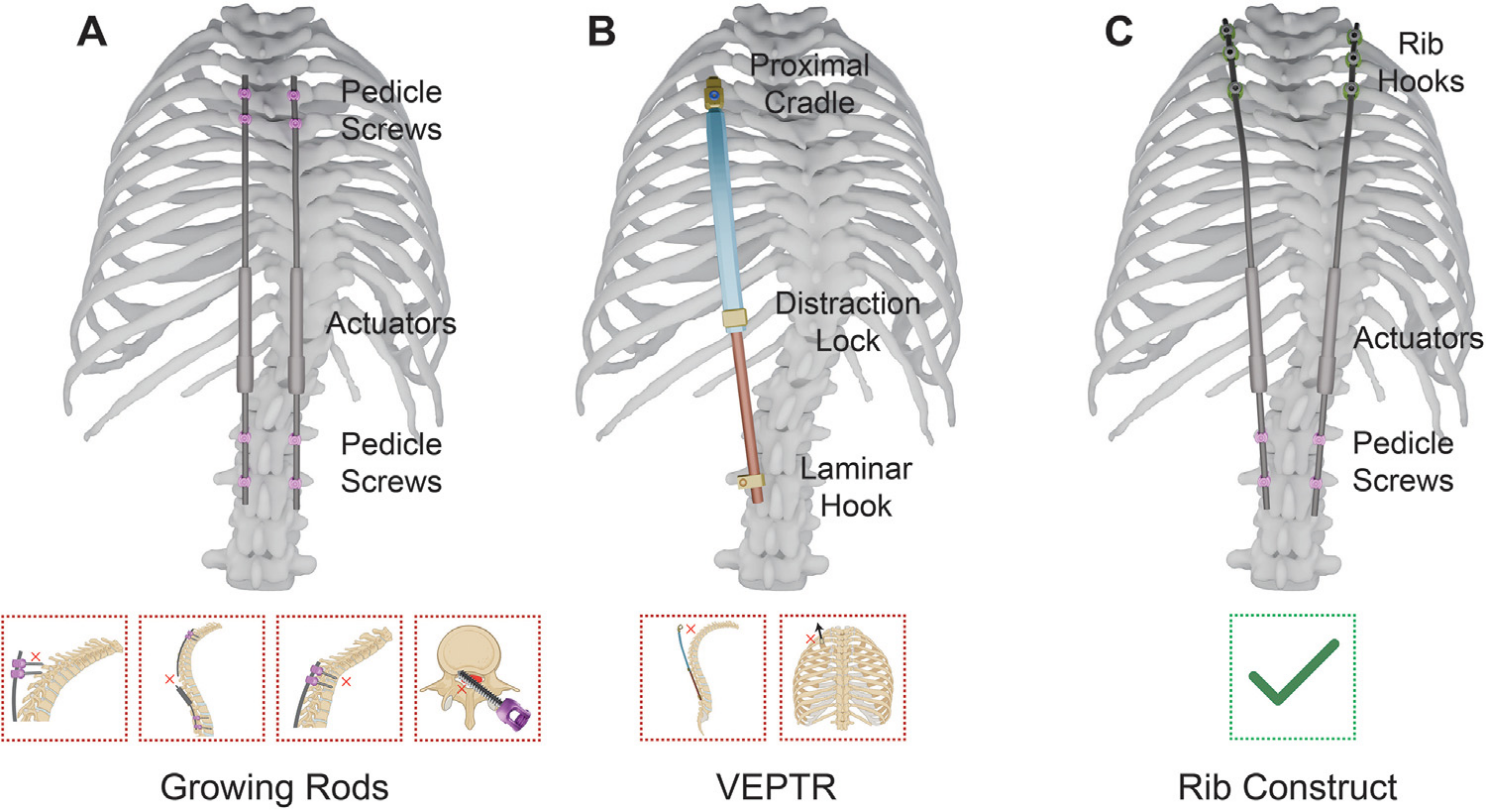
**Early-onset spinal deformity surgical management.** A) Growing rods with proximal and distal intra-spinal pedicle screw fixation and expandable connector. The growing rods are lengthened as the child grows with serial lengthening procedures. Lengthening may be achieved via manual distraction through an open incision, a spring distraction system (SDS- Cresco Spine), or a transcutaneous magnetic actuator (MAGEC- NuVasive). Common complications include proximal fixation failure with pedicle screw pull-out, rod fracture, and proximal junctional kyphosis (PJK). Neurological injury from pedicle screw invasion of the spinal canal is less common, but catastrophic when it does occur. (B) VEPTR device shown. Corrects coronal plane deformity but the majority of the implant cannot be contoured in the sagittal plane and is, thus, contraindicated in hyperkyphosis and kyphoscoliosis. VEPTR is also relatively high-profile causing implant prominence through the skin, and has only 1 or 2 narrow proximal cradles, which has led to implant migration through the rib. (C) Hybrid rib construct shown. Series of hooks anchored to bilateral rods on the proximal superior ribs for proximal fixation. Distal fixation is achieved with laminar hooks or pedicle screws to the spine, or sacral/alar hooks or iliac screws to the pelvis.

The primary goal of treatment for EOSD is to correct the deformity while promoting spinal growth and improving pulmonary function, with minimal complications [6]. The most common growth-sparing surgical treatment for EOSD is growing rods anchored with proximal and distal intra-spinal pedicle screw fixation, which are periodically lengthened to align the spine until skeletal maturity (Fig. 2A) [[7], [8], [9]]. However, this method is frequently ineffective, especially in high-risk EOSD patients with nonidiopathic etiologies or osteoporosis. The rigid nature of growing rods fixed to the spine with pedicle screws can cause auto-fusion of the vertebrae in more than 80% of patients, resulting in compromised pulmonary function and sub-optimal spinal growth [10]. Mechanical complications are rampant, ranging between 50% in low-risk patients to >200% in high-risk patients with severe deformity [[11], [12], [13], [14], [15]]. For example, proximal fixation failure with pedicle screw pull-out and rod fracture occurs in >33% of high-risk patients [11,12]. A progressive kyphosis just superior to the proximal fixation, called “proximal junctional kyphosis” (PJK) occurs in >28% of patients [13]. Finally, neurological injury from pedicle screw misplacement or migration is a rare but catastrophic complication [14,15].

An alternative growth-sparing device, called the VEPTR (Vertical Expandable Prosthetic Titanium Rib), uses rib fixation for correction of early-onset scoliosis (Fig. 2B). However, its fixed radius and inability to be contoured in the sagittal plane renders it ineffective for managing hyperkyphotic and kyphoscoliotic deformities [[16], [17], [18], [19]]. Furthermore, VEPTR is relatively high-profile, causing implant prominence through the skin, and has only 1 or 2 narrow proximal cradles, which has led to implant migration through the rib. Nevertheless, VEPTR has demonstrated that the ribs can provide strong anchor points without the need for direct spinal instrumentation. This inspired the development of a hybrid “rib construct” utilizing this favorable feature, but capable of being adapted to address any type of 3-dimensional deformity, including hyperkyphosis or kyphoscoliosis [[20], [21], [22]]. This construct employs a series of hooks anchored to bilateral growing rods on the proximal superior ribs for proximal fixation (Fig. 2C). The rib construct concept was first developed by 1 of our co-authors, Richard Gross, who published the first case report describing this technique in 2012 [20].

The rib construct represents a paradigm shift from intra-spinal pedicle screw fixation to extra-spinal, rib-based fixation that enables 3-dimensional correction of thoracic deformities. Unlike VEPTR, it can be contoured in any plane, including the sagittal plane, allowing direct manipulation of the thoracic cavity to optimize rib alignment, thoracic volume, and pulmonary function—especially in hyperkyphotic and kyphoscoliotic patients [23,24]. It is also possible the rib construct may improve spinal growth with reduced rates of auto-fusion, mechanical complications, and risk of neurological injury compared to more rigid pedicle screw-based growing rod constructs.

The work described here consists of 3 components. First, an ex vivo biomechanical study examined the ability of the rib construct to withstand a bending or torsional force and compared it to traditional pedicle screw fixation. Second, a pediatric porcine model study examined the ability of the rib construct to correct hyperkyphosis and remodel the spine. Finally, a retrospective clinical study assessed the rib construct’s ability to correct deformity in human patients. The collective results demonstrate that the rib construct is a safe and effective technique in patients with EOSD.

## Materials and methods

### Ex vivo biomechanical study

#### Specimen preparation

Twenty pig spines from C5 to L6 with intact rib cages (*n*=10 for the rib construct group, and *n*=10 for the pedicle screw group) were harvested from 8-week-old Yorkshire domestic male pigs. Pigs of this age were used because the size and weight of their spines and rib cages (spinal height 53.6±4.1cm, body weight 21.3±1.3kg) are similar to those of the pediatric human patient population. Specimens were cleaned of paravertebral soft tissues, with care taken to preserve ligamentous structures. Titanium rods (5.5mm×500mm) were instrumented bilaterally in both groups. The pig has 15 ribs, so based on the contour of the superior ribs, laminar hooks were placed on the ribs in a claw formation, with 2 down-going hooks on ribs 3 and 4 and 2 up-going hooks on ribs 5 and 6. In the pedicle screw group, pedicle screws (5.0mm×20mm) were placed in T3 and T4 bilaterally in accordance with standard technique. Proximal fixation was affixed to bilaterally contoured rods. After the spines were instrumented, the specimens were potted (Bondo Filler, 3M, Maplewood, MN) proximally and distally in custom potting fixtures. The proximal ends of the specimens were potted to T1, while the distal ends were potted to L4.

#### Mechanical testing

**Fig. 3:**
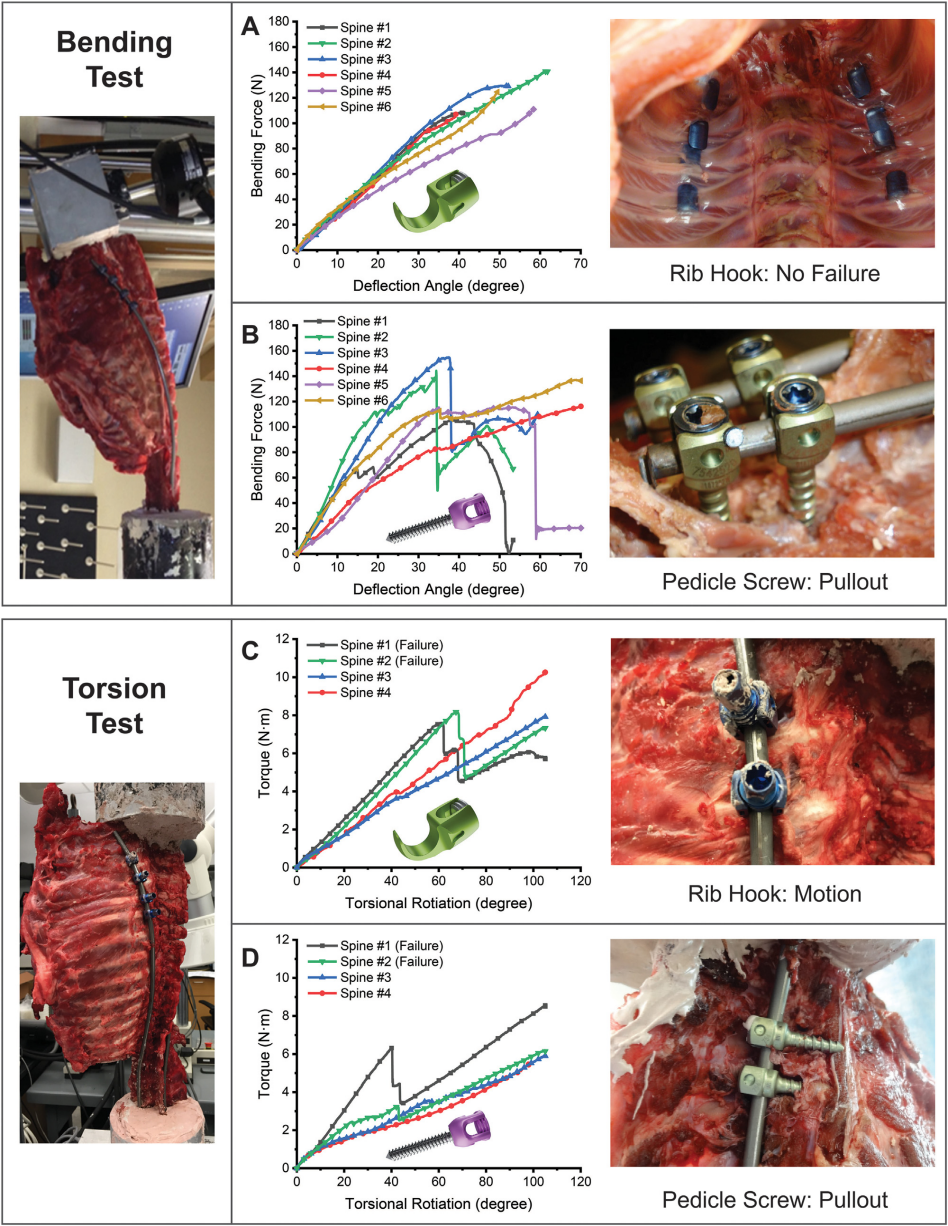
**Superior biomechanical performance of rib fixation compared to pedicle screws.** Test configuration for bending using an MTS system. Inline load cell with actuator measured bending force at the potted proximal end of each specimen. A) Bending force vs. deflection angle for each specimen in the rib construct group. No proximal fixation failure was observed for any of the 6 spines tested, with each bending test reaching the maximum spine deflection allowed by the test system. B) Bending force vs. deflection angle for each specimen in the pedicle screw group. Proximal fixation failure was recorded for all 6 spines tested for bending testing, with the failure mode being pedicle screw pull-out (arrow). C) Torque vs torsional rotation for each specimen tested in the rib construct group and D) pedicle screw group. Proximal fixation failure occurred in 2/4 specimens tested for both groups, with hook migration and pedicle screw plowing being the respective failure mechanisms.

The distal potted ends of the spines were anchored with custom mounts to the base of the mechanical testing system (858 Mini Bionix II, MTS, Minneapolis, MN). Two types of mechanical tests were performed: 1) bending testing, simulating pull-out forces on proximal instrumentation (*n*=6), and 2) torsional testing, simulating twisting forces on instrumentation (*n*=4) (Supplementaryary Fig. S1). For the bending test, a pure bending force was applied by the mechanical testing system to the proximal end [25]. For torsional testing, a rotational force was applied to the proximal end. The force (F) and deflection angle (θ) were measured by a load cell and optical tracking system, respectively, and were time synchronized. Bending testing quantified the force and deflection angle, and torsional testing quantified the torque and torsional angle applied to the spine that induced mechanical failure of the instrumentation (Fig. 3).

### Porcine animal model study

#### Hyperkyphosis creation

**Fig. 4:**
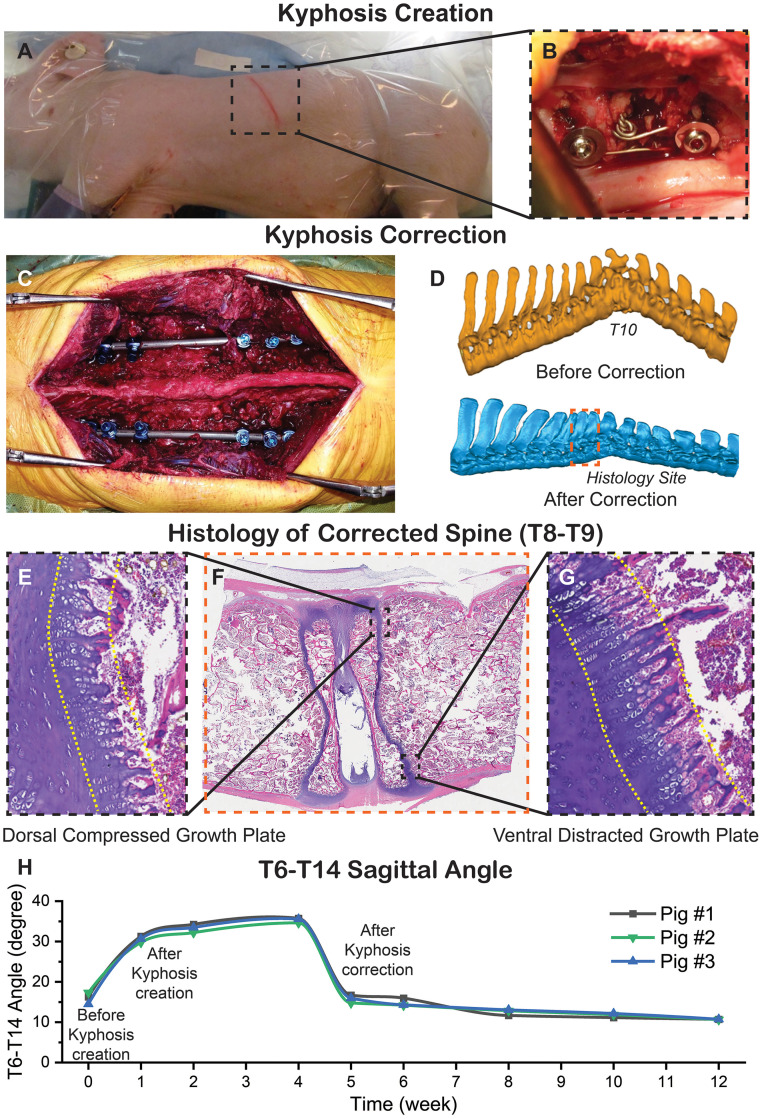
**Creation and subsequent correction of hyperkyphosis with the rib construct in a porcine early-onset spinal deformity animal model.** A) Kyphosis creation. Site of thoracotomy between ribs T10-T11. Exposure of the spine, placement of screws and partial resection of T10 vertebral body. B) Wire loop placed around screws and tightened. Kyphosis correction. C) Proximal fixation with rib construct T6-T8, hyperkyphosis apex T9-T11, distal fixation with rib construct T12-T14. D) 3-D reconstructed CT images of porcine spine before deformity correction (top) and 8 weeks after the deformity correction using the rib construct (bottom). E-G) H&E staining at T8-T9 of corrected spine. Growth plates bowed inwards, compression of posterior discs and anterior extrusion of nucleus pulposus in instrumented disc spaces of corrected spine. E) Section of dorsal T9 growth plate that was compressed with rib construct. G) Section of ventral T9 growth plate that was distracted with rib construct. Reduced cellularity, smaller chondrocyte columns in dorsal (compressed) portion of growth plate, compared to ventral (distracted) portion of growth plate. H) Radiographic imaging data. Approximately 16.0° T9-T11 Cobb angle preop, 30.6° postop, and 35.3° at follow-up prior to corrective surgery. In response to corrective surgery with the rib construct, T6-T14 thoracic hyperkyphosis decreased immediately postop to 15.8° and continued to decrease to 10.7° at final follow-up 8 weeks postop (week 12).

Our technique for creating a porcine hyperkyphotic model has been published [26]. All procedures were approved by the Institutional Animal Care and Use Committee (IACUC). Hyperkyphosis creation was performed in 6 10kg (male, 5-week-old) immature Yorkshire pigs. Procedure protocol was 1) a left thoracotomy at T10-11, 2) screw placement at T9 and T11, 3) partial vertebrectomy at T10, 4) posterior interspinous ligament transection, and 5) placement of wire loop around screws and tightening. Immediate hyperkyphosis after surgery was ∼30° between T6-T14 (∼30° T9-T11) with the apex at T10. The hyperkyphosis increased to ∼35° T6-T14 (∼40° T9-T11) at 4 weeks postop, prior to corrective surgery (Fig. 4A, B, H).

#### Hyperkyphosis correction with rib construct

Initially, distal fixation for corrective instrumentation was placed in the lumbar spine, but immediate junctional failure uniformly occurred because the lumbar spine is hypermobile, relative to the thorax [27]. Thus, a totally thoracic extraspinal construct was developed. With the hyperkyphotic deformity at T9-11, a superior claw was placed on ribs 6–8, and an inferior claw at ribs 12–14. The rods were placed without contouring, thus placing an extension moment on the deformity (Fig. 4C). Detailed procedure description for the porcine animal model study can be found in Supplementary Materials and Methods.

#### Radiographic evaluation

Sagittal fluoroscopic images of the thoracic spine were acquired using a c-arm (OEC® 9800 Plus, GE Healthcare, Chicago, IL) preop, immediately postop, and at regular intervals until the final follow-up 8 weeks after corrective surgery. With each radiograph, thoracic kyphosis was measured between T6–T14. Pigs were euthanized 8 weeks postop. At necropsy, computed tomography (CT) scans were obtained, and 3-dimensional reconstructions were generated. CT was used to measure wedging of vertebral bodies (in degrees) (Supplementary Table S1).

#### Histological evaluation

Instrumentation was removed, and spinal segments from T4–L1 were immersion-fixed in Cal-Ex II (Fisher Scientific) for fixation and decalcification. Spines were hemi-sected longitudinally. A 3.0mm thick slab section was then removed from the cut face and subsequently bisected to allow placement into tissue cassettes. Tissues were paraffin embedded, with 5mm thick sections placed on negatively charged glass slides and stained with hematoxylin and eosin (H&E). Sections were examined, and digitally reconstructed sub-gross images were captured. Growth plates were examined qualitatively for chondrocyte cellularity and column length in the anterior and posterior growth plates of instrumented spinal segments adjacent to T9–T11.

### Retrospective clinical study

#### Study design and patients

A retrospective study was conducted on 14 human patients (8 male, 6 female) that were treated with the rib construct for severe nonidiopathic spinal deformity at The Medical University of South Carolina (MUSC) between 2007 and 2015. This study examined male and female patients, and similar findings are reported for both sexes.

All 14 patients (average age 11-years-old) received the rib construct as a growth-sparing treatment for early onset spinal deformity, meaning it was lengthened as the patients grew. Number of lengthening procedures was 2.7±1.6. Upon reaching skeletal maturity, 13 of the 14 underwent definitive fusion procedures (9 with the rib construct and 4 with pedicle screws). Final follow-up was 7.3±2.5 years. This study was approved by the MUSC institutional review board (IRB). Patient demographics and procedural data can be found in Supplementary Table S2.

#### Operative technique

Full weight-bearing anteroposterior and lateral view radiographs were obtained prior to surgery. Surgeries were performed under general anesthesia with the patient in the prone position on a Jackson table. Sensory and motor neuromonitoring was used in all cases. A straight midline incision was made from T2-T5. For the initial 2 cases, a subperiosteal dissection was performed from the laminae to the proximal second to fifth ribs, leaving the midline structures intact. This was modified to perform a subcutaneous dissection laterally to the region of the proximal second to fifth proximal ribs, at which point an incision was made through the paraspinal muscles, followed by a subperiosteal exposure 1cm lateral to the costo-transverse junction on ribs 2–5. A tract was created with a laminar finder over the superior surfaces of ribs 2 and 3, and the inferior surfaces of ribs 4 and 5. Down-going hooks were placed on the superior surfaces of ribs 2 and 3 and up-going hooks on the inferior surfaces of ribs 4 and 5. After bilateral proximal rib anchor placement, distal lumbar and/or pelvic anchor fixation was placed.

Appropriate length titanium 4.5mm or 5.5mm rods were contoured. The rods were provisionally loaded superiorly, and the hooks gently compressed. Rods were then mated to distal fixation. Deformity correction in the sagittal plane was achieved by contouring the rods *in situ* with l-benders, and in the coronal plane by a combination of distraction and contouring the rods.

Instrument lengthening procedures were performed dependent on growth rate. For definitive fusion procedures in skeletally mature patients, the rib construct was left in place and crushed cancellous bone graft was placed along the transverse processes. Deformity correction was evaluated with clinical follow-up and radiographs.

#### Follow-up and data collection

Collected data included diagnosis, age at index surgery, length of follow-up, T-score bone density, complication rates, procedure time, operative blood loss, and radiographic outcomes. Average follow-up was 6 years. Specific radiographic data included preop, postop, and final follow-up sagittal and coronal Cobb angles, sagittal vertical axis (SVA), coronal vertical axis (CVA), pelvic tilt (PT), pelvic incidence (PI), sacral slope (SS), C2-C7 angle, C2-C7 sagittal vertical axis (SVA), lumbar lordosis (LL), spinal height (T1–S1), thoracic height (T1–T12), apical vertebral translation (AVT), T1 tilt, clavicle angle (CA), and total lung capacity (TLC) (Supplementary Fig S2 and S3). Total lung capacity was estimated using antero-posterior radiographs according to the established technique described by Schlesinger et al. [28]. The longitudinal distance (cm) from the apex of the right lung to the right costophrenic angle was designated “L,” while the transverse width from the right to the left costophrenic angles was designated “W”. The equation for TLC (in liters) =(0.163*L)+(0.189*W)-4.928.

### Statistical analysis

Statistical analyses included 1-way ANOVA and Tukey’s post hoc tests to determine differences in preop, postop, and final follow-up radiographic measurements, and biomechanical outcomes.

## Results

### Ex vivo biomechanical study

Mechanical testing was performed on porcine cadaver spines instrumented with the rib construct or pedicle screw fixation. Both pure bending and torsional testing were performed, and the deflection angle and force applied to specimens were measured until mechanical failure of instrumentation. For bending testing, the rib construct group had no failures, despite each bending test reaching the maximum spine deflection allowed by the testing system (Fig. 3A). In contrast, with the pedicle screw group, fixation failure occurred for all tested spines (Fig. 3B), all of which experienced pedicle screw pull-out as the failure mode.

Table 1 compares the biomechanical outcomes of the rib construct and pedicle screw groups for bending testing and demonstrates the superior performance of the rib construct. The rib construct withstood 97.9±10.0° of bending and 119.7±13.9N of maximal force with no proximal fixation failure. This was significantly greater than the pedicle screw group, which withstood 64.6±7.3° of bending (p<.001) and 118.6±25.7N of maximal force, at which point all proximal fixations failed. The average construct stiffness for the rib construct group was 2.75±0.34N/degree, which was significantly softer than the 3.58±0.78N/degree observed in the pedicle screw group (p<.05). The rib construct approach was also technically successful, as the pleura was not violated, and there were no rib fractures in any of the 6 specimens.

**Table 1:** Bending test performance of pedicle screws versus rib construct.

	Pedicle screws	Rib construct
**Proximal fixation failure**	Yes (6/6)	No (0/6)
**Initial deflection angle**	28.8±7.2	47.6±6.0*
**Total deflection angle**	64.6±7.3°	97.9±10.0°*
**Maximal force**	118.6±25.7N	119.7±13.9N
**Construct stiffness**	3.58±0.78N/degree	2.75±0.34N/degree *

mean ± standard deviation, *p<.05.

For torsional testing, fixation failure occurred in 2/4 spines tested for both the rib construct and pedicle screw groups. Hook migration on the ribs was the failure mode for the rib construct group, while pedicle screw plowing was the failure mode for the pedicle screw group. The rib construct group withstood significantly greater torque/torsion angles than the pedicle screw group (p<.05) prior to failure (Fig. 3C–D, Table 2).

**Table 2:** Torsional test performance of pedicle screws vs. rib construct.

	Pedicle screws	Rib construct
**Proximal fixation failure**	Yes (2/4)	Yes (2/4)
**Deflection angle**	46.9±9.2°	57.9±13.3° *
**Maximal Torque**	3.7±0.7N.m	6.0±2.0N.m*

mean ± standard deviation, *p<.05.

### Porcine animal model study

Hyperkyphosis was created and subsequently corrected with the rib construct without complications in 3 experimental pigs (*N*=3) (Fig. 4). Detailed surgical technique for creation (Fig. 4A, B) and correction (Fig. 4C–G) of hyperkyphotic deformity can be found in Supplementary Materials and Methods.

#### Radiographic data

Sagittal fluoroscopic images were obtained preop, immediately postop, and at regular intervals until necropsy (week 12), 8 weeks after corrective surgery (*N*=3). The preop T6–T14 thoracic kyphosis was 16.0±1.4°. The T6–T14 thoracic hyperkyphosis created at the time of initial hyperkyphosis creation surgery was 30.6±0.8° and increased to 35.3±0.6° prior to corrective surgery. In response to corrective surgery with the rib construct, T6–T14 thoracic hyperkyphosis decreased immediately postop to 15.8±1.0° and continued to decrease to 10.7±0.1° at final follow-up 8 weeks postop (p<.001) (Fig. 4H).

CT images at necropsy showed wedging, indicating that growth modulation was occurring. Wedging averaged 4.7±4.1°, 10.6±4.3°, and 1.6±2.8° in vertebral bodies T7, T8, and T12 respectively, adjacent to T9–T11 in corrected spines. Three dimensional reconstructions of a hyperkyphotic and a corrected spine are shown (Fig. 4D).

#### Histological evaluation

Histologic evaluation of spinal segments T4–L1 revealed fusion and stunted growth of the ventral growth plates between T9–T10 and T10–T11, as expected from hyperkyphotic deformity creation surgery. The dorsal growth plates in these segments remained intact and continued to grow relatively uninhibited, which is why the deformity progressed prior to corrective surgery. Growth plates and intervertebral discs in segments adjacent to T9 and T11 remained intact.

In response to correction with the rib construct, analysis of spinal segments adjacent to T9–T11 revealed realigned orientation with a concavity on the posterior side and convexity on anterior side, resulting in a straighter more anatomically aligned spine. The nucleus pulposus between instrumented segments was extruded anteriorly with compression of the posterior disc space. Cellular changes indicative of growth modulation was observed in the growth plates of instrumented spinal segments. Macroscopically, growth plates in corrected spines were bowed inwards (Fig. 4F). Qualitatively, there appeared to be reduced cellularity and column length in hypertrophic and proliferative zones of the dorsal compressed section of growth plates (Fig. 4E) compared to the ventral distracted section (Fig. 4G).

#### Rib observations

There were no rib fractures or violations of the pleura in any of the pigs treated with the rib construct. However, ossification was observed on the implant surface. This ossification was unique to the pig model and did not occur in the clinical study.

### Clinical study

#### Patient population and surgery information

A total of 14 patients that received the rib construct were studied. All 14 patients received the rib construct as a growth-sparing treatment for EOSD. There was 1 patient with isolated hyperkyphosis, 5 with scoliosis, and 8 patients that had kyphoscoliosis. Etiology was syndromic (7), neuromuscular (5), and congenital (2). All patients had osteoporosis. Bone density was T scores −4.0±1.0. Age at rib construct index surgery was 11.1±1.7 years. The procedure time was 5:00±1:30 hours for rib construct index surgery. The blood loss was 277±159mL for index surgery. Number of lengthening procedures was 2.7±1.6. Upon reaching skeletal maturity, 13 of the 14 underwent definitive fusion procedures (9 with the rib construct and 4 with pedicle screws). Final follow-up was 7.3±2.5 years.

#### Radiographic data

The rib construct effectively corrected kyphosis and scoliosis deformities in pediatric patients. Hyperkyphosis sagittal Cobb angle was 73.0° preop, 24.8° postop, and 9.3° at final follow-up. Scoliosis coronal Cobb angle was 66.1±10.5° preop, 37.6±13.3° postop, and 26.3±18.4° at final follow-up; a 60% correction (p<.001). For patients with kyphoscoliosis, coronal Cobb angle was 80.8±29.8° preop, 63.1±29.2° postop after rib construct index surgery, and 61.4±32.5 at final follow-up; a 24% correction (p<.001) (Table 3). Kyphoscoliosis sagittal Cobb angle was 91.8±23.0° preop, 39.6±21.5° postop, and 27.2±20.3° at final follow-up; a 100% correction (p<.001) (Table 4). Spinal height (T1-S1) and total lung capacity (TLC) increased in all groups. There was improved alignment of the shoulders, neck, head and thorax, supported by improvements in coronal and sagittal T1 tilt, clavicle angle (CA), sagittal vertical axis (SVA), C2–C7 angle, and C2–C7 sagittal vertical axis (SVA). A comprehensive summary of all radiographic outcomes with 95% confidence intervals can be found in Table 3, Table 4. Patient level data can be found in Supplementary Table S2–4.

**Table 3:** Growth-sparing RC treatment coronal plane radiographic outcomes.

	Hyperkyphosis (*N*=1)	Scoliosis (*N*=5)	Kyphoscoliosis (*N*=8)		Total (*N*=14)
**Coronal cobb angle**					
**Preop**	10.0°	66.1 (53.1, 79.1)°		80.8 (55.9, 105.7)°	70.5 (53.5, 87.5)°
**Postop RC**	9.0°	37.6 (21.1, 54.1)°		63.1 (38.7, 87.5)°	50.1 (33.7, 66.5)°
**Final follow-up**	8.0°	26.3 (3.5, 49.1)°		61.4 (34.2, 88.6)°	45.0 (26.1, 63.9)°
**Coronal T1 Tilt**					
**Preop**	8.8°	17.3 (2.4, 32.2)°		15.7 (7.8, 23.6)°	15.7 (10.2, 21.2)°
**Postop RC**	3.6°	7.2 (1.1, 13.3)°		5.2 (1.2, 9.2)°	5.7 (3.0, 8.4)°
**Final follow-up**	9.1°	9.0 (−0.4, 18.4)°		4.4 (0.8, 8.0)°	6.2 (3.0, 9.4)°
**Coronal vertical axis (CVA)**					
**Preop**	2.2 cm	2.3 (0.3, 4.3) cm		2.4 (0.7, 4.1) cm	2.4 (1.4, 3.4) cm
**Postop RC**	1.6 cm	2.0 (0.8, 3.2) cm		2.5 (0.7, 4.3) cm	2.3 (1.3, 3.3) cm
**Final follow-up**	3.4 cm	2.4 (−0.6, 5.4) cm		1.8 (0.6, 3.0) cm	2.1 (1.1, 3.1) cm
**Apical vertebral translation (AVT)**					
**Preop**	0.0 cm	4.3 (0.7, 7.9) cm		6.2 (2.4, 10.0) cm	5.1 (2.7, 7.5) cm
**Postop RC**	0.0 cm	2.2 (1.2, 3.2) cm		3.6 (1.9, 5.3) cm	2.9 (1.8, 4.0) cm
**Final follow-up**	0.0 cm	2.4 (1.2, 3.6) cm		4.3 (2.5, 6.1) cm	3.4 (2.1, 4.7) cm
**Clavicle angle (CA)**					
**Preop**	4.1°	8.6 (−0.3, 17.5)°		7.9 (5.2, 10.6)°	7.8 (5.2, 10.4)°
**Postop RC**	8.1°	3.4 (−0.4, 7.2)°		3.4 (0.7, 6.1)°	3.8 (2.0, 5.6)°
**Final follow-up**	4.7°	3.3 (−0.4, 7.0)°		2.9 (0.7, 5.1)°	3.2 (1.8, 4.6)°
**Spinal height (T1-S1)**					
**Preop**	29.8 cm	29.4 (24.9, 33.9) cm		29.9 (26.7, 33.0) cm	29.7 (27.7, 31.7) cm
**Postop RC**	34.5 cm	32.7 (26.4, 39.0) cm		35.1 (31.9, 38.3) cm	34.2 (31.8, 36.6) cm
**Final follow-up**	39.0 cm	34.6 (28.9, 40.3) cm		37.3 (34.0, 40.6) cm	36.5 (34.1, 38.9) cm
**Thoracic spinal height (T1-L1)**					
**Preop**	20.4 cm	18.3 (15.3, 21.3) cm		17.6 (14.7, 20.5) cm	18.1 (16.4, 19.8) cm
**Postop RC**	22.1 cm	20.2 (17.1, 23.3) cm		20.6 (17.5, 23.7) cm	20.6 (18.8, 22.4) cm
**Final follow-up**	24.8 cm	21.5 (20.1, 22.9) cm		23.3 (20.3, 26.3) cm	22.7 (21.0, 24.4) cm
**Total lung capacity (TLC)**					
**Preop**	1.3 L	1.6 (0.5, 2.7) L		1.6 (0.8, 2.4) L	1.6 (1.0, 2.2) L
**Postop RC**	1.3 L	1.7 (0.7, 2.7) L		1.6 (0.9, 2.3) L	1.6 (1.1, 2.1) L
**Final follow-up**	3.0 L	2.6 (1.7, 3.5) L		2.5 (1.7, 3.3) L	2.6 (2.1, 3.1) L

mean (95% confidence interval).

**Table 4:** Growth-sparing RC treatment sagittal plane radiographic outcomes.

	Hyperkyphosis (*N*=1)	Scoliosis (*N*=5)	Kyphoscoliosis (*N*=8)	Total (*N*=14)
**Sagittal cobb angle**				
**Preop**	73.0°	33.9 (28.1, 39.7)°	91.8 (72.6, 111.0)°	69.8 (50.7, 88.9)°
**Postop RC**	24.8°	18.1° (10.4, 25.8)°	39.6 (21.6, 57.6)°	30.9 (19.7, 42.1)°
**Final follow-up**	9.3°	21.9 (5.8, 38.0)°	27.2 (10.2, 44.2)°	24.0 (14.0, 34.0)°
**Sagittal T1 Tilt**				
**Preop**	15.7°	27.5 (15.6, 39.4)°	51.5 (33.9, 69.1)°	40.4 (28.7, 52.1)°
**Postop RC**	12.0°	24.3 (13.9, 34.7)°	30.2 (18.5, 41.9)°	26.8 (19.6, 34.0)°
**Final follow-up**	12.8°	22.2 (13.1, 31.3)°	23.7 (18.7, 28.7)°	22.4 (18.6, 26.2)°
**Sagittal vertical axis (SVA)**				
**Preop**	1.0 cm	2.3 (−2.4, 7.0) cm	7.6 (2.8, 12.4) cm	5.6 (2.4, 8.8) cm
**Postop RC**	3.1 cm	3.7 (0.1, 7.3) cm	2.0 (−1.3, 5.3) cm	2.6 (0.5, 4.7) cm
**Final follow-up**	−3.8 cm	1.1 (−4.2, 6.4) cm	4.2 (2.9, 5.5) cm	3.0 (1.2, 4.8) cm
**Pelvic tilt (PT)**				
**Preop**	13.4°	19.5 (−2.6, 41.6)°	30.2 (16.8, 43.6)°	24.8 (15.3, 34.3)°
**Postop RC**	1.8°	20.5 (3.0, 38.0)°	26.8 (18.4, 35.2)°	22.2 (14.8, 29.6)°
**Final follow-up**	20.3°	18.0 (9.6, 26.4)°	22.3 (11.9, 32.7)°	20.6 (14.9, 26.3)°
**Pelvic incidence (PI)**				
**Preop**	45.1°	55.2 (45.8, 64.6)°	48.0 (36.0, 60.0)°	50.3 (43.5, 57.1)°
**Postop RC**	41.1°	50.7 (37.9, 63.5)°	47.8 (40.9, 54.7)°	48.3 (43.3, 53.3)°
**Final follow-up**	48.6°	45.5 (35.6, 55.4)°	45.9 (34.5, 57.3)°	46.0 (39.9, 52.1)°
**Sacral slope (SS)**				
**Preop**	29.6°	37.6 (20.2, 55.0)°	28.4 (18.3, 38.5)°	31.8 (24.6, 39.0)°
**Postop RC**	42.2°	31.4 (21.6, 41.2)°	22.1 (13.0, 31.2)°	27.3 (20.9, 33.7)°
**Final follow-up**	22.9°	28.8 (19.7, 37.9)°	29.5 (22.6, 36.4)°	28.6 (24.4, 32.8)°
**C2-C7 angle**				
**Preop**	–	–	34.7 (18.1, 51.3)°	–
**Postop RC**	–	–	16.0 (3.4, 28.6)°	–
**Final follow-up**	–	–	9.4 (2.0, 16.8)°	–
**C2-C7 sagittal vertical axis**				
**Preop**	–	–	2.8 (1.9, 3.7) cm	–
**Postop RC**	–	–	2.3 (1.9, 2.7) cm	–
**Final follow-up**	–	–	2.1 (1.4, 2.8) cm	–
**Lumbar lordosis (LL)**				
**Preop**	67.5°	50.7 (29.6, 71.8)°	34.0 (5.7, 62.3)°	43.7 (27.9, 59.5)°
**Postop RC**	59.8°	33.4 (22.7, 44.1)°	35.4 (22.9, 47.9)°	36.6 (28.8, 44.4)°
**Final follow-up**	42.0°	37.4 (29.5, 45.3)°	34.8 (24.5, 45.1)°	36.5 (31.1, 41.9)°

mean (95% confidence interval).

#### Complications

The group as a whole was medically fragile, representing the extreme spectrum of severe deformity and poor bone quality. There was a total of 24 complications. There were 16 complications during growth-sparing treatment with the rib construct, with a complication rate of 0.0, 0.2, and 1.88 complications per patient in the hyperkyphosis, scoliosis, and kyphoscoliosis groups, respectively. There were 8 complications postdefinitive fusion in the entire study, with a complication rate of 0.0, 0.4, and 0.75 complications per patient in the hyperkyphosis, scoliosis, and kyphoscoliosis groups, respectively. The most common complications during growth-sparing treatment were rod fracture (4), iliac set screw failure (3), hook dislodgement (2), rib fracture (2), and sacral rod migration (2). Rod fractures occurred at a rate of 0.0, 0.2, and 0.38 per patient in the hyperkyphosis, scoliosis, and kyphoscoliosis groups, respectively. The most common complications post definitive fusion were pseudarthrosis (3), rod fracture (2), and prominent implant discomfort (2). One pseudarthrosis followed a repeat insertion of a rib construct placed 1 year after removal for a delayed deep wound infection. A second pseudarthrosis occurred under a synovial cyst overlying the thoracic spine, which blocked any posterior vasculature of the fusion mass. There was no proximal junctional kyphosis (PJK), no changes in intra-operative neuromonitoring, no complications that induced neurological deficits, and no intercostal neurovascular injury in any patients during growth-sparing treatment and post definitive fusion. A comprehensive list of complications can be found in Table 5.

**Table 5:** Growth-sparing RC treatment complications.

	Hyperkyphosis (*N*=1)	Scoliosis (*N*=5)	Kyphoscoliosis (*N*=8)	Total (*N*=14)
**Rod fracture**				**Total=6**
**During growth-sparring**	0	1	3	4
**Postdefinitive fusion**	0	0	2	2
**Pseudarthrosis**				**Total=3**
**During growth-sparring**	–	–	–	–
**Postdefinitive fusion**	0	0	3	3
**Prominent implant discomfort**				**Total=3**
**During growth-sparring**	0	0	1	1
**Postdefinitive fusion**	0	2	0	2
**Iliac set screw failure**				**Total=3**
**During growth-sparring**	0	0	3	3
**Postdefinitive fusion**	0	0	0	0
**Sacral rod migration**				**Total=2**
**During growth-sparring**	0	0	2	2
**Postdefinitive fusion**	0	0	0	0
**Iliac screw pull-out**				**Total=1**
**During growth-sparring**	0	0	1	1
**Postdefinitive fusion**	0	0	0	0
**Hook dislodgement**				**Total=2**
**During growth-sparring**	0	0	2	2
**Postdefinitive fusion**	0	0	0	0
**Rib fracture**				**Total=2**
**During growth-sparring**	0	0	2	2
**Postdefinitive fusion**	0	0	0	0
**Infection**				**Total=2**
**During growth-sparring**	0	0	1	1
**Postdefinitive fusion**	0	0	1	1

### Case examples

#### Hyperkyphosis

**Fig. 5:**
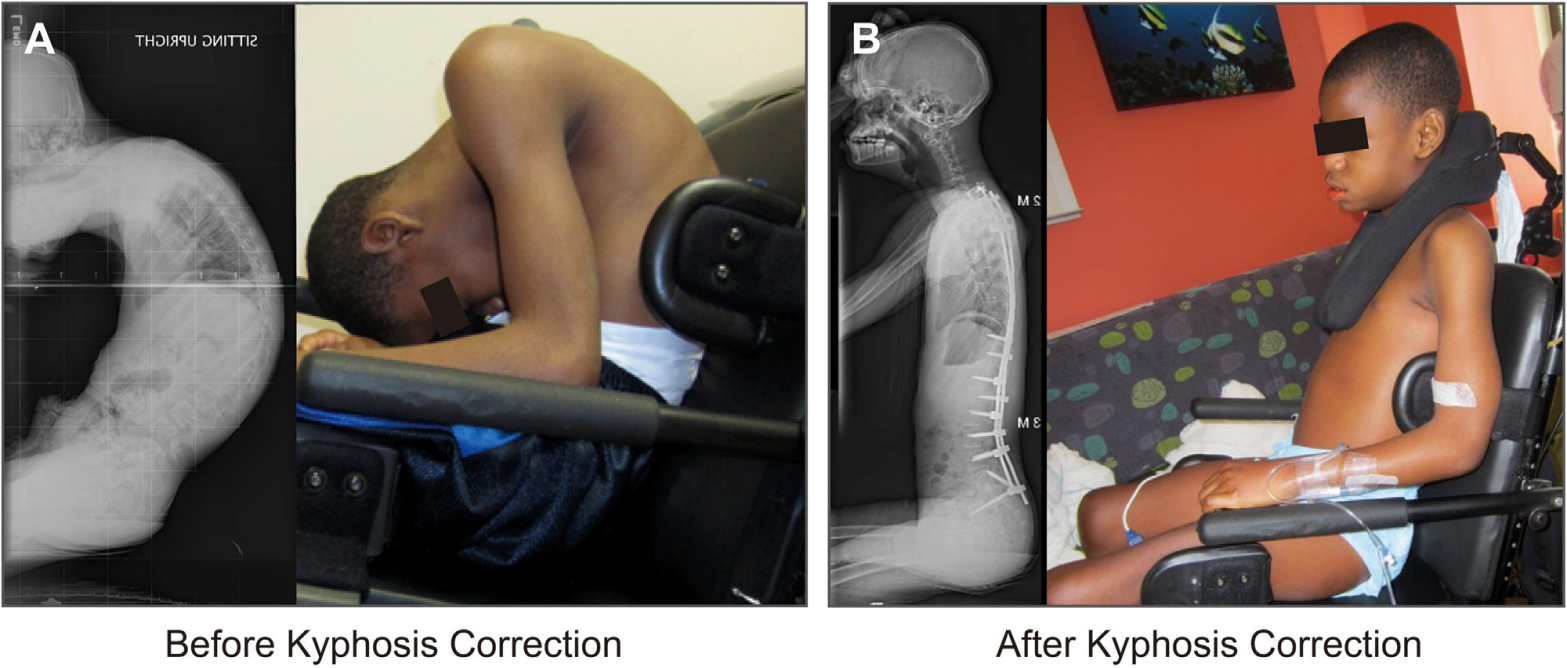
**Early-onset hyperkyphosis correction with rib construct.** A) An 11-year-old boy with spastic quadriparesis had severe kyphoscoliosis, including a global hyperkyphotic deformity of 130°. B) A rib construct was placed. His hyperkyphosis was completely corrected, and he was able to sit up in his chair. Final thoracic kyphosis measured 32°.

An 11-year-old boy with spastic quadriparesis had severe kyphoscoliosis, including a global hyperkyphotic deformity of 130° with his head positioned towards his groin area (Fig. 5). He was severely osteoporotic with a T score −4.6. A rib construct was placed. His hyperkyphosis was completely corrected, and he was able to sit up in his chair. This patient did not receive dedicated construct lengthening procedures. However, he was allowed to grow for an additional 15 months prior to definitive fusion. Final thoracic kyphosis measured 32°. Qualitatively, his work of breathing, activities of daily living, and quality of life were substantially improved compared to preoperatively.

#### Scoliosis

**Fig. 6:**
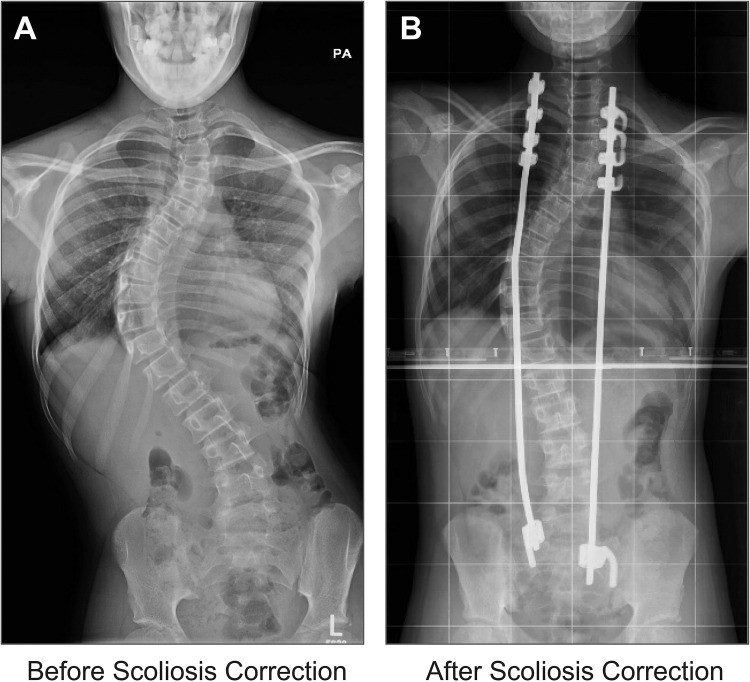
**Early-onset scoliosis correction with rib construct.** A) A 10-year-old girl with 71° thoracic scoliosis. B) She was treated with a growth-sparing rib construct, which corrected her scoliosis to 39°.

A 10-year-old girl with 71° thoracic scoliosis (Fig. 6) had rapidly progressing deformity, back pain, and functional limitations. A rib construct was placed, with immediate correction of thoracic scoliosis to 39°. One year later, the patient was approaching skeletal maturity, and fusion with pedicle screw construct was performed. Final thoracic scoliosis was 22°, an acceptable correction. The patient was pain free and had no complications.

#### Kyphoscoliosis

**Fig. 7:**
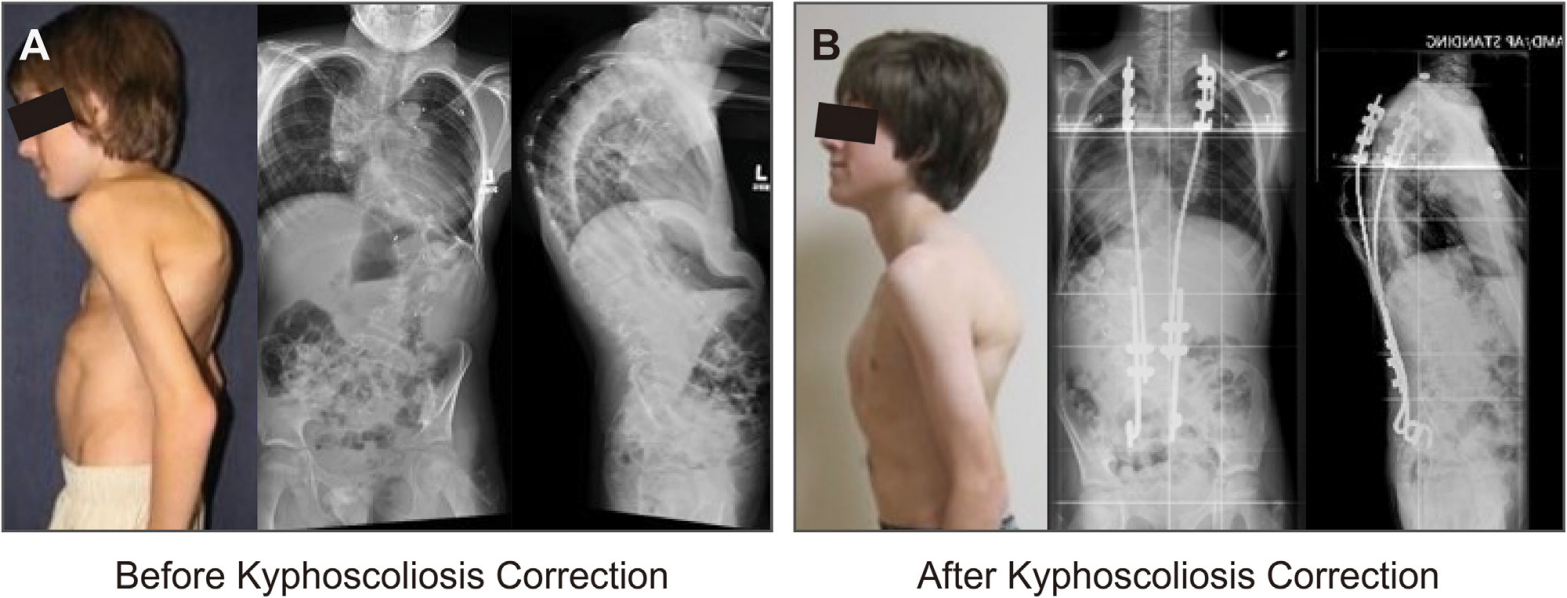
**Early-onset kyphoscoliosis correction with rib construct.** A) A 12-year-old boy with VATER Syndrome and 73° thoracic scoliosis, 72° thoracolumbar scoliosis, and 102° thoracic hyperkyphosis. B) He was treated with a growth-sparing rib construct, which completely corrected his hyperkyphosis, greatly improved his scoliosis, and leveled his shoulders and pelvis. Five lengthening procedures were performed. Definitive fusion with pedicle screw construct at skeletal maturity.

A 12-year-old boy with VATER Syndrome had a 70° thoracic curve at age 5 and had rapid deterioration at age 12 with severe rigid kyphoscoliosis (Fig. 7). His preoperative radiographic measurements were 73° thoracic scoliosis, 72° thoracolumbar scoliosis, and 102° thoracic hyperkyphosis. He had progressive osteoporosis with a T score −3.9. A previous pedicle screw construct failed and was not tolerated by the patient. He was treated with a growth-sparing rib construct, which completely corrected his hyperkyphosis, greatly improved his scoliosis, and leveled his shoulders and pelvis. Five lengthening procedures were performed over 4 years with the rib construct. Once skeletal maturity was reached, definitive fusion was performed with a pedicle screw construct.

## Discussion

### Clinical relevance

EOSD is a substantial global public health problem. Treatment is complex, with high complication rates even in skilled hands. In areas with sparse resources, children are not treated and subject to a reduced life span secondary to the pulmonary deficits associated with severe spinal deformity. Even in resource-rich areas, treatment of EOSD is problematic. There is a lack of consensus among spine surgeons regarding management. We propose a redirection of emphasis from spinal correction with intra-spinal fixation, to thoracic realignment using extra-spinal rib fixation. The goal is to manage the deformity while promoting a well-developed thoracic cavity, improved lung volume, and improved pulmonary function with minimal complications. Results from this study support the value of the rib construct as a safe and effective method that achieves this goal. Our clinical and translational science investigations present strong evidence that the rib construct can achieve this goal effectively.

### The rib construct effectively corrects spinal deformity

Clinical results indicate that growth-sparing treatment with the rib construct can correct kyphosis and scoliosis deformities. Radiographic evidence demonstrated superior hyperkyphosis correction and similar scoliosis correction, compared to growing rods with pedicle screws and VEPTR [10,16]. In addition to improvements in sagittal and coronal Cobb angles, other parameters of trunk, shoulder, neck and head alignment improved as well. Since the rib construct allows the surgeon to manipulate the whole thorax as a unit, rather than just the spine, the rib construct is highly effective at correcting trunk, shoulder, neck and head malalignment. This conclusion was supported by notable improvements in coronal and sagittal T1 tilt, clavicle angle (CA), sagittal vertical axis (SVA), C2–C7 angle, and C2–C7 sagittal vertical axis (SVA).

### The rib construct supports spinal growth and pulmonary development

Patients with EOSD often have associated deformities of the rib cage and thorax that result in compromised pulmonary function [29]. Correction of the spinal deformity with spine-based instrumentation usually has an attenuated and incomplete correction of the rib cage deformity. Previous studies have documented minimal change in stereoradiographic modeling of the rib cage after growing rod procedures [30]. For example, correction of rib cage rotational deformity is only 2/3 that of spinal correction [31]. By directly manipulating the rib cage, the rib construct addresses rib cage deformity and provides more lung space. In hyperkyphotic patients, the rib construct can pull the proximal ribs posteriorly and elevate the thorax into a more mechanically favorable position for ventilation, similar to the function of the accessory muscles of respiration. This is especially important for patients who have compromised respiratory function due to generalized muscle weakness [23]. This study documented improvements in total lung capacity in hyperkyphotic patients, measured by radiographs, after treatment with the rib construct. Measurements were made radiographically and must be regarded as preliminary. However, it is clear that thoracic posture was improved postop.

The stiff nature of growing rods fixed to the spine with pedicle screws often causes auto-fusion of the vertebrae in >80% of patients, resulting in diminishing returns with each lengthening procedure, stunted spinal growth, and compromised pulmonary function [10]. Because the rib construct uses extra-spinal fixation to the ribs, it avoids spinal fusion of fixation sites and auto-fusion of other levels, thus maximizing natural spinal growth and pulmonary development. The mobile nature of the costovertebral joint also reduces the need for frequent lengthening procedures. Rib-based fixation is less rigid than spine-based fixation with pedicle screws and allows more spinal growth between lengthening procedures. In this series of patients, only 2.7±1.6 lengthening procedures were necessary per patient, approximately 1 procedure every 12 months until skeletal maturity. This is compared to 1 procedure every 6-8 months, typically performed with growing rods with pedicle screw fixation. Excellent improvement in spinal height (T1-S1) and thoracic spinal height (T1-L1) were observed with the rib construct. No auto-fusion was observed in any patient.

### Incidences of the most troublesome complications associated with treatment of early-onset spinal deformity were substantially reduced with the rib construct

Compared to growing rods with proximal pedicle screw fixation, the rib construct offers several advantages that reduce risk of complications. The rib construct has more bone-implant interface with strong cortical bone, allowing for higher corrective forces to be safely applied with reduced risk for proximal fixation failure. The less rigid nature of rib fixation and preservation of important posterior midline ligaments may also reduce the risk of rod fracture and PJK. Since the rib construct does not require manipulation of structures close to the thoracic spinal cord, the failure mode of rib fixation is safer than intra-spinal fixation, and the risk of neurological injury is minimized.

The clinical study group was medically fragile, with etiologies and severity of deformity associated with high complication rates [32]. Therefore, although this series is small, it represents the most challenging portion of the spectrum of severity for EOSD. The reported incidence of proximal junctional kyphosis (PJK) resulting from treatment is around 25–28% with both growing rods and VEPTR [13,33,34]. There was a notable lack of PJK with the rib construct. In fact, the chin brow angle was improved in 3 patients with pre-existing PJK following failed prior surgery with pedicle screw fixation. Critically, by manipulating the thorax instead of the spine, the important posterior midline spinal ligaments remain intact and act as a tether. When hyperkyphosis is corrected, these preserved ligaments pull the head and neck into a more upright position, resulting in substantially improved forward positioning of the head. By completely avoiding the posterior ligaments during exposure and avoiding placement of spinal stress risers, the rib construct can essentially eliminate the PJK complication.

The most devastating complications of treatment for early-onset spinal deformity are neurologic. A recent multicenter study of treatment for severe deformity with complex osteotomies by expert surgeons revealed a 44% incidence of neuromonitoring changes, and a 9% incidence of new neurologic deficits [35]. In our series, 14 of 24 patients would qualify for the criteria described in this previous multicenter study. Notably, none of our patients had neuromonitoring changes or new neurologic deficits, underscoring the safety of the rib construct. The avoidance of neurological complications was likely because the rib construct does not require manipulation of structures close to the thoracic spinal cord.

### The rib construct is especially useful in cases that involve hyperkyphosis and/or osteoporosis

Proximal fixation failure with pedicle screw pull-out is 1 of the most common mechanical failures in EOSD patients, especially with hyperkyphosis and osteoporosis [11]. This complication occurs in >33% of these high risk patients, compared to only 14% (2/14) in this rib construct study [11]. This is due to high stress on the pedicle screws causing them to pull out of the bone. Compared to growing rods with pedicle screw fixation, the rib construct has more bone-implant interface with strong cortical bone, while pedicle screws have limited interface and with weaker cancellous bone. As a result, much higher corrective forces can be safely applied by the surgeon using the rib construct, especially in the sagittal plane, without the potentially disastrous effects of pedicle screw failure. This mechanical mechanism was supported by mechanical testing and clinical data in the present study. Ex vivo biomechanical results documented that the rib construct provides stronger proximal fixation that is less prone to failure and less stiff than pedicle screw fixation, when subjected to bending forces simulating hyperkyphosis, and torsional forces. Porcine spines instrumented with the rib construct were less prone to failure compared to pedicle screws and were able to withstand greater bending force/torque and deflection/torsional angle. Rod fracture is also a common problem with growing rods, especially in patients with hyperkyphosis. Previous studies have demonstrated rod fracture rates in growing rods with pedicle screws exceeding 25% for early-onset scoliosis (EOS) and >150% for EOSD with concurrent hyperkyphosis [6,11]. Rod fractures in this rib construct study were 20% (1/5) during growth-sparring treatment for EOS and 37.5% (3/8) for EOSD with hyperkyphosis. This is likely due to the stiff nature of pedicle screw fixation, resulting in high stress in the rods. In the rib construct, motion at the costovertebral joints makes the rib construct less stiff than growing rods, reducing the risk of rod fracture.

### The rib construct may reduce the need for halo gravity traction and osteotomy techniques, such as vertebral column resection

The higher corrective forces that can be safely applied with the rib construct provide a form of temporary “portable traction”, reducing the need for halo gravity traction and osteotomies in definitive fusion cases. Halo gravity traction is a technique that can be used preoperatively, where traction is applied to the spine through a metal ring (halo) that is attached to the patient’s head [36]. This is typically used for patients with large rigid curves, with the goal of stretching out the spine slowly over a period of weeks prior to surgery. Osteotomies of the posterior spinal structures are currently being performed with increasing frequency for all types of pediatric deformity [[37], [38], [39]]. Although generally safe in experienced hands, they are associated with increased blood loss, neuromonitoring changes, and possible neurological injury [40]. Vertebral column resection is a particularly invasive osteotomy technique that is widely utilized as a corrective measure for spinal deformity. We show that the use of the rib construct may lessen the need for halo gravity traction, vertebral column resection, and other types of osteotomies. Halo gravity traction and osteotomies were not used for any patients in this series, indicating that substantial correction is possible without their use. In fact, the rib construct can be used as a form of “portable traction” in patients with large rigid curves to partially correct deformities prior to undergoing definitive fusion. The simplicity of the rib construct technique, combined with the ability to avoid halo gravity traction and complex osteotomy procedures, makes this technique especially valuable for enhancing access to treatment in developing nations.

### The rib construct corrects spinal deformity by growth modulation

Porcine model results validated the safety and effectiveness of the rib construct. This animal model elucidated the mechanobiological mechanism by which the rib construct and other growth-sparing techniques correct early-onset spinal deformity. The rib construct provides some immediate deformity correction by realigning vertebral bodies. Additional correction is gradually achieved over time via growth modulation [41]. Instrumentation can be strategically applied to take advantage of this process to straighten a deformed spine. The principle behind this is referred to as the “Hueter Volkmann Law,” which states that tensile forces stimulate longitudinal bone growth, while compressive forces inhibit growth [42]. The present study found wedging of vertebral bodies in response to instrumentation with the rib construct. Histology showed macroscopic bowing of growth plates and qualitatively reduced cellularity and chondrocyte column length in the dorsal compressed sections of growth plates compared to ventral distracted sections. While previous studies have used porcine models to study scoliosis, this was the first study to create and subsequently correct hyperkyphotic spinal deformity in a skeletally immature animal model and examine the outcomes at the sub-gross and cellular level [43,44].

### Limitations and opportunity for future innovation

One limitation of this study is that at the time this research began, there were no hooks available on the market that were specifically designed for rib fixation. This work was performed with off-label use of spinal hooks which are designed for spinal fixation to the lamina but were used on the ribs during the development of this surgical technique. Our group filed the first intellectual property in 2015 for hooks designed specifically for rib fixation [45]. Since then, numerous other rib hook systems have become available from companies including NuVasive (alternative thoracic fixation), Highridge (universal hooks), and Orthopediatrics (Response Rib and Pelvic System). Despite tremendous progress, there are still opportunities to improve the design of these rib hook systems by lowering the implant profile, enhancing contact area with the rib, and improving the ease with which they can be implanted with minimally-invasive techniques.

Another limitation of this study is the relatively small sample size for the clinical study, and this was not a randomized controlled clinical trial. However, a small sample size is appropriate and necessary for preliminary data to support safety and efficacy. There was no control group with pedicle screws for growing rods or VEPTR. Most of the patients treated with the rib construct were challenging cases, and 4 had previously been unsuccessfully treated with growing rods and pedicle screws. There has never been a published prospective randomized controlled clinical trial for the treatment of EOSD, and this would be an excellent follow-up study to conduct. Despite these limitations, these data clearly demonstrate that the rib construct is safe and effective. The rib construct may be a substantial advancement in the treatment of children with spinal deformity around the globe.

## Conclusions

The rib construct effectively corrects spinal deformity through growth modulation while supporting spinal growth and pulmonary development. It substantially reduces the incidence of severe complications commonly associated with EOSD treatments and is particularly beneficial in cases involving hyperkyphosis and/or osteoporosis. Additionally, the rib construct may decrease the need for invasive techniques such as halo gravity traction and vertebral column resection. Dissemination of this technique could improve outcomes for EOSD patients across the globe.

## Statements and declarations

None.

## Ethical considerations

The retrospective clinical study was conducted under IRB protocol Pro00082303, titled “Rib Construct for Correction of Early Onset Spinal Deformity (EOSD).” The complementary animal study was conducted under IACUC protocol IACUC-2020-01122-1, titled “Studies on the Effect of the Rib Construct on Growth Modulation of a Fixed Kyphotic Spine Deformity in a Porcine Model”.

## Consent to participate

Not applicable.

## Consent to publication

Not applicable.

## Funding statement

Scoliosis Research Society research grant (RG)

NIH predoctoral fellowship TL1TR001451 (DB)

NIH predoctoral fellowship F31AR076917 (DB)

NIH grant P20GM121342 (HY)

NIH grant UT2GM130174 (HY)

NIH Phase I SBIR R43AR080507 (HY)

NIH Phase II SBIR R44AR080507 (DB, HY)

## Data availability

The data in this study will be made available on reasonable request to correspondence authors through email.

## A short summary sentence

The rib construct, an alternative technique for correcting early-onset spinal deformity (EOSD), was evaluated through biomechanical testing, an animal model, and retrospective clinical data, demonstrating effective deformity correction with fewer complications.

## Declaration of competing interest

The authors (DB, HY) of this paper have interest in Apex Orthopaedic Technologies. Pertinent IP includes US Patent No. 10,441,321 titled “Rib Hook Devices, Systems, and Methods of Use” issued on October 15, 2019 (inventor: DG, GW, HY).

## Appendix Supplementary materials

Image, application 1
