# Supplementary material for: Rib construct for severe spinal deformity in young children: a 3-part investigation of biomechanical, animal, and clinical case data
Source: N Am Spine Soc J. 2025 Jul 30;23:100776. doi: 10.1016/j.xnsj.2025.100776 (PMC12444494; doi:10.1016/j.xnsj.2025.100776)
Supplement: Supplementary file 1 [file mmc1.docx]

Supplementary Materials for: **Rib Construct for Severe Spinal Deformity in Young Children: A 3-Part Investigation of Biomechanical, Animal, and Clinical Case Data**

**The content:**

Materials and Methods

Fig. S1. Mechanical testing schematics.

Fig. S2. Clinical study coronal plane radiographic measurements.

Fig. S3. Clinical study sagittal plane radiographic measurements.

Table S1. Porcine model radiographic data.

Table S2. Patient demographics and procedural data.

Table S3. Patient coronal and sagittal Cobb angle data.

Table S4. Patient complications data.

**Materials and Methods**

**Detailed Procedure Description for Porcine Animal Model Study**

*Hyperkyphosis creation*

1. Preoperative feeding was limited to a liquid diet prior 24 hours prior to surgery.

2. The animal was anesthetized via injectables followed by isoflurane intubation. Preemptive use of analgesia was performed.

3. The thoracic region of the porcine was shaved and the skin cleaned with 7.5% povidone-iodine and 70% isopropyl alcohol and the surgical site appropriately draped.

4. Dorsoventral and lateral fluorographs of the spine were made to document the baseline information.

5. Right-side anteriolateral thoracotomy (5 cm midline incision between T9-11, right lateral position, initial plan) was done to expose distal thoracic spine, as no dissection of diaphragm was necessary.

6. Holes were be drilled into T9 and T11 and surgical cancellous bone screws (4mm diameter) and washers (Synthes Inc) were inserted. The positioning of the screws were verified by fluoroscopy.

7. Once the screws were implanted, a rongueurs surgical instrument was used to remove the anterior and anterior lateral cortices of the center vertebral body, allowing anterior compression and creation of kyphotic deformity. This procedure took about 5-10 minutes.

8. Mersilene suture (0), or 18-20 gauge stainless steel sterile wire was used to create a tether between the two cancellous screws.

9. A dorsal midline incision measuring 4-6 cm was made to allow release of the dorsal interspinous ligament over the manipulated vertebral body to allow kyphotic curve to the spine.

10. The wound was closed in layers. The muscle and skin incision was closed. The incision site was covered with sterile dressing for draining until the porcine returned to their houses (cages).

11. Our post-operative analgesia treatment was given pre-emptively and 24 hours after surgery then PRN every 12 hrs. The wound was observed for infection daily for the first week and every other day thereafter.

12. Animals were assessed by fluorography on a weekly basis.

13. Animals were allowed to survive kyphosis surgery for 3-5 weeks and then either underwent a second surgery to implant the rib construct to modulate spinal growth (or sham control) or will have no further treatment but survived an equal amount of time (3-8 weeks) to serve as implant device controls.

*Hyperkyphosis correction with rib construct*

After a kyphotic deformity of at least 35 degrees (Cobb angle) was established between T9-T11, a rib construct was implanted in the spine for growth modulation. The rib construct incorporated three thoracic ribs proximal to the kyphotic deformity (T6-8) and three thoracic ribs distal to the kyphotic deformity (T12-14). The pigs were then be followed until skeletal maturity. The spine was assessed radiographically at weekly intervals for remodeling.

The step by step clinical procedures included:

1) Preoperative feeding was limited to a liquid diet prior 24 hours prior to surgery.

2) The animal was anesthetized using ketamine and preemptive analgesia was be delivered.

3) The animal was intubated and placed on a respirator, and electrocardiogram and a temperature leads were attached. IV lines were placed in the ear vein for delivery of crystaloid fluids and antibiotics.

4) The the entire back region of the porcine was shaved and the skin cleaned with 7.5% povidone-iodine and 70% isopropyl alcohol and the surgical site appropriately draped.

5) Dorsoventral and lateral radiographs of the spine were made to document the baseline information.

6) After prone positioning on a grounding pad, a shave of the thoracic and lumbar region, sterile prep, and drape were completed.

7) A subperiosteal exposure was performed about 1 cm lateral to the transverse processes on ribs 6-8 and contuse down to T12-15. The process was repeated for the transverse side and the lumbar region was clamped together to avoid heat loss.

8) The ribs were counted forward to ensure proper construct attachment to ribs attached to T6-8 and T12-14. Muscle layers were blunt dissected away from the ribs.

9) A tract was developed with the laminar finder. Two down-going hooks will be placed on the superior aspects of T6-7 ribs, and an up-going hooks was placed in the inferior aspects of the T8 rib. The process was repeated with up-going hooks placed on T13-14 ribs and a down-going hook on T12 rib.

10) A rod was placed and the up-going hooks were gently compressed against the down-going hooks to create stable fixation.

11) By using the same technique, another rib construct was placed bilaterally.

12) The thoracic tissues were re-supported by passing #1 or 2 PDS suture through the spinous processes into the thoracic tissue of each side. This was provide along with the muscle and skin layer closures.

13) Local analgesia was delivered to the tissues in thoracic region and the wounds were closed in layers (muscle layers and skin). The incision site was covered with sterile dressing for draining until the porcine return to house (cages).


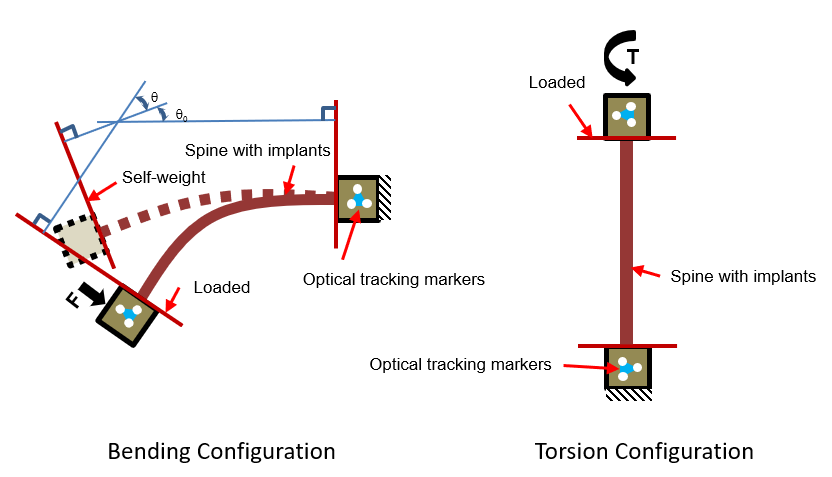


**Fig. S1. Mechanical testing schematics. A)** Test configuration for bending using an MTS system. Inline load cell with actuator measured bending force at potted proximal end of specimen. The bending force (*F*) depicted by arrow, and deflection angle ($\theta$) were measured by the load cell and tracking markers, respectively. **B)** Test configuration for torsion using an MTS system. Load cell with actuator measured torque at potted proximal end of specimen. The torque (*T*) depicted by arrow, and deflection angle ($\theta$) were measured by the load cell and tracking markers, respectively.


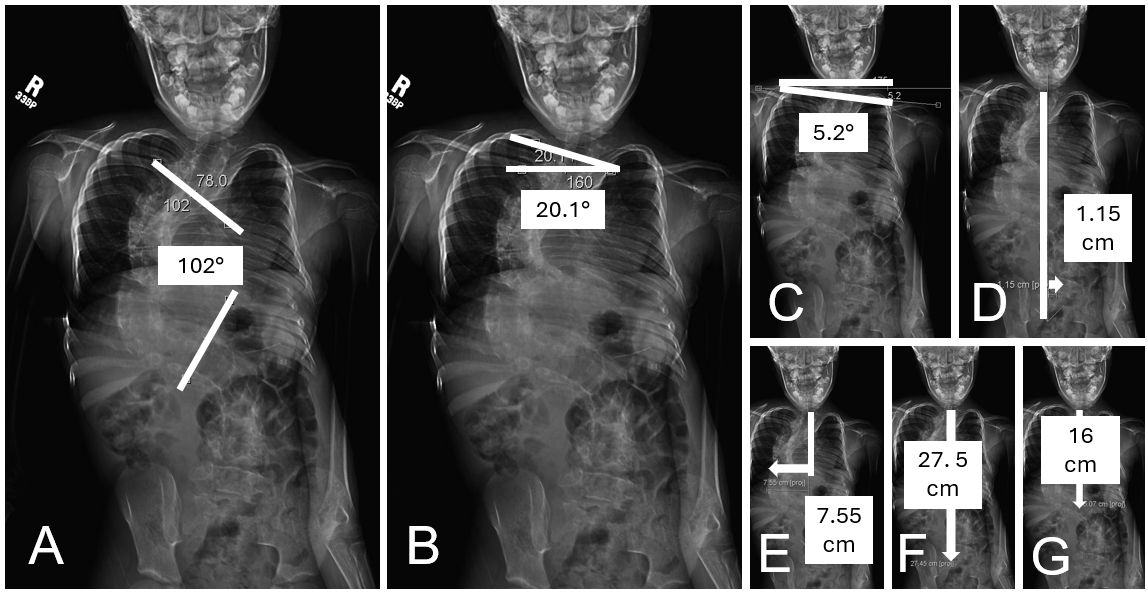


**Fig. S2. Clinical study coronal plane radiographic measurements. A)** Coronal Cobb angle, **B)** Coronal T1 tilt, **C)** Clavicle angle, **D)** Coronal vertical axis, **E)** Apical vertebral translation, **F)** Spinal height, **G)** Thoracic height.

**
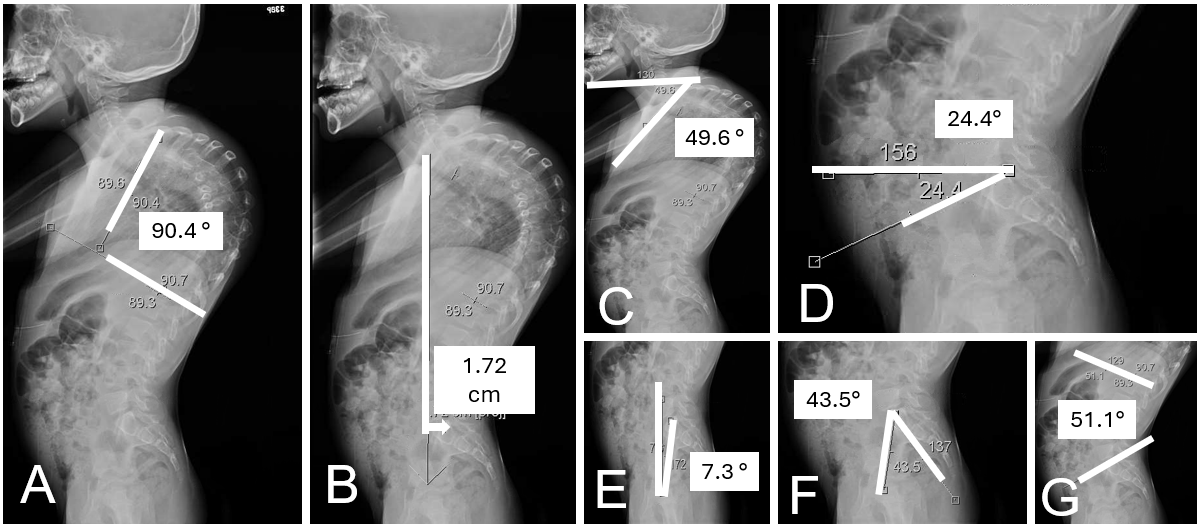
**

**Fig. S3. Clinical study sagittal plane radiographic measurements. A)** Sagittal Cobb angle, **B)** Sagittal vertical axis, **C)** Sagittal T1 angle, **D)** Sacral slope, **E)** Pelvic tilt, **F)** Pelvic incidence, **G)** Lumbar lordosis.

**Table S1. Porcine model radiographic data**

|  | **Pre-kyphosis Creation** | **Post-kyphosis Creation** | **Week 2** | **Week 4** | **Post-kyphosis Correction** | **Week 2** | **Week 4** | **Week 6** | **Week 8** |
| --- | --- | --- | --- | --- | --- | --- | --- | --- | --- |
| Pig 1 | 16.28 | 31.27 | 34.24 | 35.74 | 16.69 | 15.94 | 11.65 | 11.11 | 10.7 |
| Pig 2 | 17.3 | 29.77 | 32.2 | 34.62 | 14.76 | 14.19 | 12.82 | 11.85 | 10.61 |
| Pig 3 | 14.47 | 30.63 | 33.48 | 35.55 | 15.96 | 14.3 | 13.04 | 12.14 | 10.72 |
| Avg | 16.02 | 30.56 | 33.31 | 35.3 | 15.8 | 14.81 | 12.5 | 11.7 | 10.68 |

**Table S2. Patient demographics and procedural data**

| **Patient** | **Etiology** | **Age of RC Index Surgery** | **T Scores** | **Procedure Time** | **Blood Loss (mL)** | **Follow up months** |
| --- | --- | --- | --- | --- | --- | --- |
| **Growth-sparing EOSD, Kyphoscoliosis** | | | | | | |
| 1 | Syndromic | 12+9 | -3.9 | not available | not available | 124 |
| 2 | Syndromic | 8+2 | -2.8 | 4:51 | 125 | 108 |
| 5 | Syndromic | 13+1 | -3.8 | 4:21 | 250 | 80 |
| 6 | Neuromuscular | 10+0 | -4.4 | not available | 250 | 74 |
| 7 | Syndromic | 12+6 | -2.9 | not available | 400 | 102 |
| 14 | Neuromuscular | 14+0 | -6 | 4:49 | 200 | 26 |
| 19 | Neuromuscular | 11+2 | -4.6 | 4:33 | 175 | 44 |
| 20 | Neuromuscular | 11+5 | -4.5 | 4:44 | 150 | 14 |
| **Growth-sparing EOSD, Scoliosis** | | | | | | |
| 8 | Neuromuscular | 8+9 | -4.3 | not available | 250 | 74 |
| 10 | Congenital | 11+7 | -5.3 | 4:00 | 200 |  |
| 23 | Syndromic | 10+4 | not available | 4:20 | 300 | 13 |
| 24 | Syndromic | 11+0 | not available | 4:45 | 150 | 72 |
| 25 | Syndromic | 11+6 | -2.8 | 4:06 | 700 | 37 |
| **Growth-sparing EOSD, Kyphosis** | | | | | | |
| 16 | Congenital | 9+3 | -3 | 8:39 | 450 | 59 |

**Table S3. Patient coronal and sagittal Cobb angle data**

| **Patient** | **Preop Coronal Cobb** | **Postop Coronal Cobb** | **Final Coronal Cobb** | **Preop Sagittal Cobb** | **Postop Sagittal Cobb** | **Final Sagittal Cobb** |
| --- | --- | --- | --- | --- | --- | --- |
| **Growth-sparing EOSD, Kyphoscoliosis** | | | | | | |
| 1 | 64 | 64 | 78 | 103 | 62.4 | 34 |
| 2 | 106 | 94 | 94 | 102 | 56.6 | 44 |
| 5 | 74 | 41.5 | 43 | 83 | 11 | 10 |
| 6 | 68 | 55.4 | 50 | 104 | 53.8 | 51 |
| 7 | 83 | 57.4 | 54 | 66 | 22.4 | -6 |
| 14 | 104 | 55.2 | 40.1 | 64.7 | 47.9 | 42.9 |
| 19 | 26.7 | 22.1 | 15.8 | 133 | 53 | 32.4 |
| 20 | 121 | 115 | 116 | 79 | 10 | 9 |
| **Growth-sparing EOSD, Scoliosis** | | | | | | |
| 8 | 62 | 55.4 | 56.5 | 30 | 13 | 7 |
| 10 | 50.7 | 18.5 | 6 | 30 | 10 | 9 |
| 23 | 71 | 39.3 | 22.84 | 32 | 22.9 | 30.1 |
| 24 | 78.7 | 40.6 | 22.8 | 40.7 | 21 | 27.5 |
| 25 | 68 | 34 | 23.4 | 37 | 23.5 | 35.8 |
| **Growth-sparing EOSD, Kyphosis** | | | | | | |
| 16 | 10 | 9 | 8 | 73 | 24.8 | 9.3 |

**Table S4. Patient complications data**

| **Patient** | **Number of growth-sparing complications** | **Growth-sparing complications** | **Number of definitive fusion complications** | **Definitive fusion complications** |
| --- | --- | --- | --- | --- |
| **Growth-sparing EOSD Kyphoscoliosis** | | | | |
| 1 | 3 | prominent implant discomfort | 0 | none |
|  |  | sacral rod migration |  |  |
| 2 | 3 | infection | 1 | rod fracture/pseudarthrosis |
|  |  | rod fracture |  |  |
|  |  | Rib fracture/hook dislodgement |  |  |
| 5 | 1 | iliac set screw failure | 2 | rod fracture/pseudoarthrosis |
|  |  |  |  | loose connector |
| 6 | 2 | hook dislodgement | 0 | none |
|  |  | rib fracture |  |  |
| 7 | 4 | sacral rod migration | 2 | pseudarthrosis |
|  |  | iliac set screw failure |  | infection |
|  |  | iliac screw pullout |  |  |
|  |  | rod fracture |  |  |
| 14 | 0 | none | 0 | none |
| 19 | 1 | rod fracture | 0 | none |
| 20 | 1 | iliac set screw failure | 0 | none |
| **Growth-sparing EOSD, Scoliosis** | | | | |
| 8 | 1 | rod fracture | 0 | none |
| 10 | 0 | none | 0 | none |
| 23 | 0 | none | 0 | none |
| 24 | 1 | prominent implant discomfort |  |  |
| 25 | 0 | none | 1 | prominent implant discomfort |
| **Growth-sparing EOSD, Kyphosis** | | | | |
| 16 | 0 | none | 0 | none |
